# Analysis of Cyclist’s Drag on the Aero Position Using Numerical Simulations and Analytical Procedures: A Case Study

**DOI:** 10.3390/ijerph17103430

**Published:** 2020-05-14

**Authors:** Pedro Forte, Daniel A. Marinho, Pantelis T. Nikolaidis, Beat Knechtle, Tiago M. Barbosa, Jorge E. Morais

**Affiliations:** 1 Department of Sports, Higher Institute of Educational Sciences of the Douro, 4560-708 Penafiel, Portugal; morais.jorgestrela@gmail.com; 2 Departamento de Desporto e Educação Física, Instituto Politécnico de Bragança, 5300-253 Bragança, Portugal; barbosa@ipb.pt; 3 Research Center for Sports Health and Human Development, 6201-001 Covilhã, Portugal; marinho.d@gmail.com; 4 Department of Sports Sciences, University of Beira Interior, 6201-001 Covilhã, Portugal; 5 School of Health and Caring Sciences, University of West Attica, 12243 Athens, Greece; pademil@hotmail.com; 6 Institute of Primary Care, University of Zurich, 8091 Zurich, Switzerland; beat.knechtle@hispeed.ch

**Keywords:** cycling, drag, comparison, CFD, analytical procedures

## Abstract

Background: Resistance acting on a cyclist is a major concern among the cycling fraternity. Most of the testing methods require previous training or expensive equipment and time-consuming set-ups. By contrast, analytical procedures are more affordable and numerical simulations are perfect for manipulating and controlling inputs. The aim of this case study was to compare the drag of a cyclist in the aero position as measured using numerical simulation and analytical procedures. Methods: An elite male cyclist (65 kg in mass and 1.72 m in height) volunteered to take part in this research. The cyclist was wearing his competition gear, helmet and bicycle. A three-dimensional model of the bicycle and cyclist in the aero position was obtained to run the numerical simulations. Computational fluid dynamics (CFD) and a set of analytical procedures were carried out to assess drag, frontal area and drag coefficient, between 1 m/s and 22 m/s, with increments of 1 m/s. The t-test paired samples and linear regression were selected to compare, correlate and assess the methods agreement. Results: No significant differences (*t* = 2.826; *p* = 0.275) between CFD and analytical procedures were found. The linear regression showed a very high adjustment for drag (R^2^ = 0.995; *p* < 0.001). However, the drag values obtained by the analytical procedures seemed to be overestimated, even though without effect (d = 0.11). Conclusions: These findings suggest that drag might be assessed using both a set of analytical procedures and CFD.

## 1. Introduction

Cycling is one of the world’s most popular sports [1]. In competitive cycling events, arrival time is the main performance variable and several strategies have been used to improve it [2]. It is possible to identify a set of propulsive forces (produced by the lower limbs on the crank) and resistive forces (drag and rolling resistance) in cycling [3]. The propulsive forces applied by the cyclist enable the translation of the cyclist-bicycle system, whereas drag and rolling resistance are the main resistive forces [4,5,6]. The final arrival time depends on the average velocity achieved to travel the distance of a given race event. Therefore, to reach faster average velocities, cyclists are keen to enhance the propulsive forces and concurrently to minimize the resistive forces [3,4]. Helmets, apparel and different body postures have been studied to minimize drag resistance, specifically pressure drag, which is the main resistive force at speeds above 5 m/s [5,7,8,9].

It is possible to find in the literature different methods to assess drag in cycling, such as analytical procedures, coasting deceleration techniques, wind tunnel testing and numerical simulations using computational fluid dynamics (CFD) [10]. The latter is widely used because it is possible to better control environmental variables such as wind and temperature. CFD provides details of the flow field surrounding the body under analysis, such as pressure drag, viscous drag, total drag, drag coefficient and pressure distributions [5,10,11]. However, most cyclists do not have ready access to wind tunnel testing or CFD. To assess drag using analytical procedures, a set of assumptions or estimations of the frontal surface area and drag coefficient are required [10]. The drag coefficient is assumed to be invariant across different speeds when assessed using analytical procedures [12]. However, it is not known how much the drag coefficient may vary at different speeds. Usually, CFD studies assess a representative participant of a specific cohort group. Moreover, this methodology avoids confounding factors such as between-subject variability [10,13]. This standard procedure running CFD analysis has also been reported early on in other studies in cycling [5,11]. Cyclists might be aware that drag variations can influence the total resistance force they are under and their training intensity [10]. Moreover, environmental conditions such as air temperature and humidity may affect the cyclist’s performance, energy cost and exercise response [10].

Several authors have argued that analytical procedures are a feasible alternative to assess drag [3,4]. Indeed, analytical procedures are more affordable in comparison to other techniques available [14,15]. Analytical procedures provide insights in real time and on-site, over a race event or training session [14]. That said, analytical procedures require estimation of the drag coefficient and the frontal surface area [14,15]. These variables have been consistently reported as the main challenges to diminishing the bias output by such procedures [14]. As far as our understanding goes, no study can be found in the literature comparing drag in cycling assessed using CFD and a set of analytical procedures; however, several authors [3,4,5,9,10] have used each method individually to assess cyclists’ drag. Thus, it is yet unclear if data collected by both procedures can be interpreted interchangeably.

The aim of this case study was to compare cyclist drag in the aero position using numerical simulation and a set of analytical procedures. It was hypothesized that in assessing and describing the same phenomenon, a bias might exist between the two techniques.

## 2. Materials and Methods

### 2.1. Sample

An elite male cyclist with 55.0 kg of body mass and 1.72 m of height volunteered to take part in this research. The participant was wearing his competition gear (jersey: 100% polyester; shorts: polyamide, polypropylene and elastane fibers) and time-trial helmet (LAS, Cronometro) and riding a road bicycle (KTM, Revelator Master 2017, 7 kg of mass). All the procedures were in accordance with the Helsinki declaration and informed written consent was obtained beforehand. Approval was granted by the Ethics Committee of the University of Beira Interior under the registration number D1608.

### 2.2. Scanning

**Figure 1:**
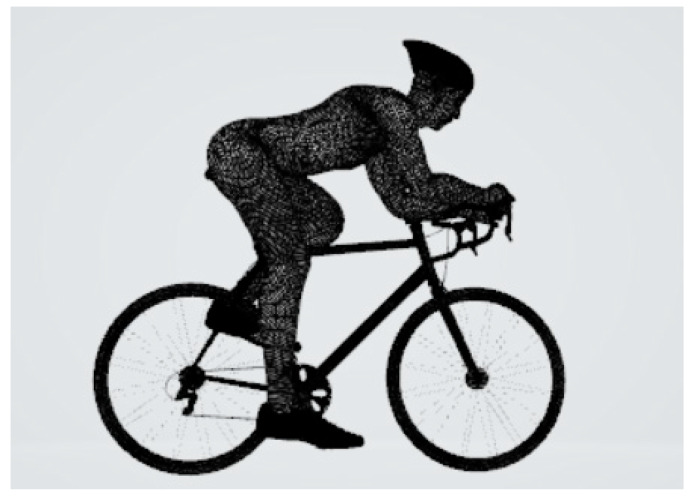
Three-dimensional model of the cyclist-bicycle system.

A three-dimensional scan (3D Systems, Inc., Rock Hill, SC, USA) was used to digitize the bicycle and the cyclist. The scanner precision was 0.0009 m (0.9 mm) at a 0.5 m (50 cm) distance, capturing spikes and roughness. The geometry was obtained with the participant in one of the most-used aerodynamic positions [6]. The geometries were edited on the Geomagic Studio software (2013, 3D System, Rock Hill, SC, USA) (Figure 1). In Geomagic, the models were corrected, removing spikes, double faces, merge parts and fill holes. Upon that, an exact organic surface was generated (3D Systems, Rock Hill, SC, USA) and converted into a Computer Aided Design (CAD) model [15].

### 2.3. Boundaries

An ASUS (ASUS, N751, Taipé, Taiwan) machine running on an Intel processor was used (Core i7 4720HQ 2,6GHz). The Central Process Unit (CPU) had 4 cores and 8 threads, a maximal turbo boost of 3.6 GHz and a speed of 5 GT/s. The computer had 12 Gb of Random Access Memory (RAM) memory and an Solid-State Drive (SSD) hard disk of 256 Gb.

On the Ansys Workbench software (Ansys Fluent 16.0, Ansys Inc., Pennsylvania, PA, USA), three-dimensional frontiers were generated as a domain around the model (domain: 7 m in length, 2.5 m in height and 2.5 m in width; model: placed at 2.5 m distance of the inlet end). The mesh was created with more than 42 million elements [16]. The elements were the volumes in which equations of motion were applied around the geometry [11,13]. The cell size was ~25 µm [11]. The mesh processing time was about 12 h.

The numerical simulations to assess drag were run between 1 m/s and 22 m/s, with increments of 1 m/s. Typically, during downhill or sprinting events, cyclists may reach the top speeds selected in this study [17,18]. Thus, each speed was set in the inlet portion of the domain (-z direction). The turbulence intensity was set as 1 × 10%−6%, and the system was set with the scalable walls function. Each computation took about 48 h to reach the simulation’s convergence.

### 2.4. Numerical Simulations

The Fluent CFD numerical code (Ansys Fluent 16.0, Ansys Inc., Pennsylvania, PA, USA) uses the finite volumes approach method to solve the Reynolds-averaged Navier–Stokes (RANS) equations. For that, a turbulence model is required, and the Realizable k-e was selected. This model was used with low-Reynolds number modelling (LRNM) to deal with the viscosity-affected region. This model presented higher convergence stability in comparison to standard k-e. Moreover, the Realizable k-e turbulence model presented a higher computation economy and velocity histograms very similar to the standard k-e, RST and RNG k-e models [15,18].

For pressure-speed coupling, the SIMPLE algorithm was used. The pressure, convection terms and viscosity were defined as second. The least squares cell-based technique allowed us to compute the gradients. Pressure and moment were defined as second and first order upwind. The turbulence kinetic energy and dissipation rate were set as first order upwind. For all the simulations, an automatic convergence occurred before 1404 interactions (Ansys Fluent 16.0, Ansys Inc., Pennsylvania, PA, USA).

### 2.5. Analytical Procedures

Drag was computed by Equation (1):

(1) $$F_{D}=0.5\rho AC_{d}v^{2}$$

where F_D_ is drag force, *ρ* is the air density (1.292 kg/m^3^), A is the surface area, estimated as 0.269 m^2^ by Equation (2) [19], and Cd is the drag coefficient, estimated as 0.733 by Equation (3) [12,20]:

(2) $$A_{p}=0.0293h^{0.725}m^{0.425}+0.0604$$

where h is the subject height and m the body mass.

(3) $$C_{d}=4.45m^{-0.45}$$

### 2.6. Statistical Analysis

Descriptive statistics, Kolmogorov–Smirnov and Levene’s tests were selected to assess normality and homogeneity. The drag value distributions for the 22 velocities for each method were tested by the Kolmogorov–Smirnov test. The t-test paired samples compared the two methods (CFD vs. analytical procedures) as in previous studies [14]. The Cohen’s d effect size was set as without effect if d < 0.2, moderate effect if 0.8 > d ≥ 0.2 and strong effect if d > 0.5 [21].

Simple linear regression models using CFD and analytical procedures were computed for the dataset in SI units (i.e., untransformed units) and after logarithmic transformation (log–log, transformed units). The determination coefficient was computed (R^2^). Effect sizes were set as very weak if R^2^ < 0.04, weak if 0.04 ≤ R^2^ < 0.16, moderate if 0.16 ≤ R^2^ < 0.49, high if 0.49 ≤ R^2^ < 0.81 and very high if 0.81 ≤ R^2^ < 1.0 [13,14].

## 3. Results

**Figure 2:**
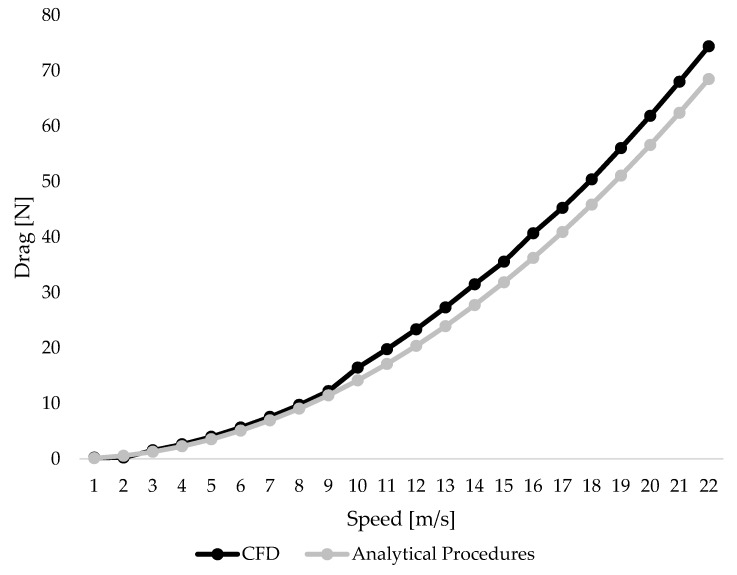
Drag values across different speeds as measured using CFD and analytical procedures.

The drag values collected using CFD ranged between 0.21 N and 74.39 N whereas with analytical procedures they ranged between 0.14 N and 68.49 N. The drag coefficient was estimated using the analytical procedures as 0.773; conversely, with CFD it ranged between 0.61 and 0.95. From 3 m/s to 22 m/s, the drag values collected using CFD were higher in comparison to analytical procedures. Figure 2 depicts the drag values collected using CFD (black line) and analytical procedures (grey line).

**Figure 3:**
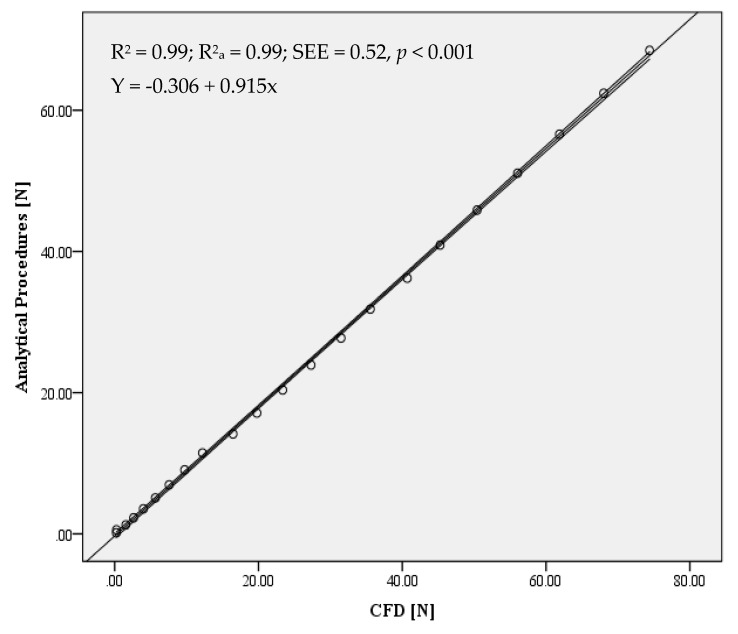

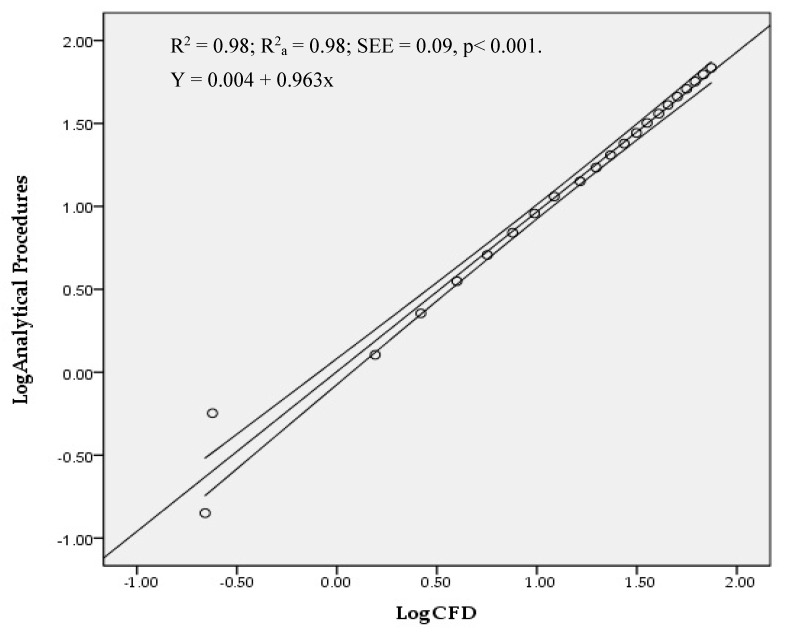
Scattergrams showing limits of agreements and trend lines in analytical procedures and CFD for absolute values (top panel) and after log–log transformation (bottom panel).

The comparison between the two techniques presented no significant differences and small effect sizes (*t* = 0.209; *p* = 0.650; *d* = 0.11). The linear regression models produced using CFD and analytical procedures presented a significant relationship and very high effect sizes for drag both in absolute units (R^2^ = 0.99; R^2^a = 0.99; SEE = 0.52; *p* < 0.001) and after log–log transformation (R^2^ = 0.98; R^2^a = 0.98; SEE = 0.09; *p* < 0.001) (Figure 3).

When the model trend line was forced to cross the axis origin (i.e., c = 0, so y = m.x), the adjustment remained as in absolute values (R^2^ = 0.99; SEE = 0.51; *p* < 0.001; Equation (4):

(4) Y=0.914x

## 4. Discussion

The aim of this study was to assess a cyclist’s drag in the aero position using numerical simulations and analytical procedures. The hypothesis was that there might be a bias between methods. The main results of this research were that the analytical procedures presented no significant differences from the numerical simulations. Moreover, the methods presented a significant and very high relationship. Nevertheless, as speed increased, drag as assessed with analytical procedures was underestimated in comparison to CFD.

CFD has been selected in several other studies to assess drag in cycling [9,16,22]. This technique is accurate when compared to wind tunnel testing [22,23,24]. Some reported an overestimation of 7%–11% in CFD results as compared to wind tunnel testing [5]. Analytical procedures enable the production of on-site and real-time data for cyclists [14]. Little is known about the different methods for assessing drag in sport settings. However, analytical procedures seem to be prone to overestimating the aerodynamics in comparison to CFD [13]. CFD has also presented an 18% underestimation in comparison to experimental testing in swimming [25]. That was justified by the impossibility for the swimmer to keep a perfectly streamlined position over the entire trial [25]. In our study, analytical procedures also underestimated drag in comparison to CFD. That might be explained by: (1) the drag coefficient being estimated and assumed as invariant across the different speeds, independently of the bicycle shape, helmets or sports gear, or (2) the frontal surface area being derived from an estimation by a mathematical model. However, studies have reported a strong agreement between data on the frontal area and the surface area [12,20,26].

The CFD drag output was 27.02 (±23.78) N and in the analytical procedures it was 24.41 (±21.76) N. The mean difference between techniques was 9%. No statistical differences, and trivial effect sizes, were found between techniques (*t* = 0.209; *p* = 0.650; *d* = 0.11). In our study, the drag coefficient as measured with analytical procedures was estimated as 0.773, and with CFD it varied between 0.61 and 0.95. To date, no study has compared drag in cycling using CFD and analytical procedures. However, it is possible to find at least one study comparing the two methods in swimming [13]. Barbosa et al. [13] noted a very high relationship between the absolute values and those after logarithmic transformation (R^2^ = 0.98; *p* < 0.001 and R^2^ = 0.99; *p* < 0.001, respectively), as in this study in cycling (R^2^ = 1.00; *p* < 0.001 and R^2^ = 0.98; *p* < 0.001, respectively). Log–log transformation has been used to minimize the distribution variance effect on linear regression [13,14].

CFD has been seen as an alternative method to the gold standard for measurement (wind tunnel) when compared to experimental techniques or analytical procedures [22,24]. On the other hand, analytical procedures have also been used in cycling [3,26] as a quick and straightforward technique to assess drag. Barbosa et al. [13] suggested that the biggest challenge to forecasting drag in swimming using analytical procedures is the prediction of the drag coefficient as an input variable. In our study, the variables that were estimated using analytical procedures were the drag coefficient and the frontal surface area whereas CFD assessed drag coefficient, surface area and drag force using numerical simulations. Defraye et al. [5] noted that CFD might overestimate the effective surface area (i.e., ACd) in comparison to wind tunnel. Thus, analytical procedures might have a larger bias compared to a gold-standard technique (e.g., wind tunnel). Based on the literature [3,13,22,23,24,26], drag coefficient variations across different velocities may explain the differences between methods.

The current study presented no significant differences between the use of CFD and analytical procedures to assess an elite male cyclist. However, a meaningful effect between methods was observed. We found a good fit between methods. The drag values obtained using analytical procedures were overestimated in comparison to CFD. The following can be considered as limitations of this research: (1) only one participant was evaluated and was only representative of other cyclists of an elite level, and (2) one single position was analyzed (i.e., the aero position). This study could help cyclists, coaches, support staff and researchers to be aware that analytical procedures can provide insights into drag force. However, drag values assessed using analytical procedures might be overestimated and as such should be corrected using the correction factor that is modelled and reported here. Follow-up projects could compare different testing methods (e.g., CFD, coasting deceleration, analytical procedures and wind tunnel) and positions and develop an analytical equation to assess drag coefficient in the function of speed at different positions. However, the most analyzed variables are drag and effective surface area [3,13,26].

In this study, drag coefficient estimation was dependent on the subject´s mass and surface area as predicted by his height [12,20]. In field and lab settings, within- and between-subject variability can affect drag estimation. Nevertheless, in our study, the analytical procedures’ estimations and the three-dimensional model for CFD were based on the same participant.

Most cyclists do not have ready access to wind tunnel testing or CFD. Wind tunnel testing requires highly-trained staff and is an expensive procedure. CFD also requires highly-trained staff to run the numerical simulations and it is a time-consuming procedure. Thus, end-users such as cyclists and practitioners should consider using the set of analytical procedures reported here to estimate drag force. That said, they should also be aware of the bias between testing techniques and the need to correct it. Altogether, this study supports the use of analytical procedures as a valid method to assess drag in comparison to CFD.

## 5. Conclusions

In conclusion, based on this case study, drag in cycling can be assessed using both a set of analytical procedures and CFD. Even though there were no significant differences and effect sizes were trivial, the analytical procedures overestimated drag force in comparison to CFD, and this should be corrected. Based on this case, coaches, athletes, support staff and researchers should be aware that in elite cyclists drag can be assessed by using either analytical procedures or CFD, choosing the most convenient at any given time.

## References

[1] Kräuter N., Lösing S., Bauer G., Schwering L., Seuter M. (2016). Supporting safety in cycling groups using LED-augmented gestures. Proceedings of the 2016 ACM International Joint Conference on Pervasive and Ubiquitous Computing.

[2] Lucia A., Earnest C., Arribas C. (2003). The Tour de France: A physiological review. Scand. J. Med. Sci. Sports.

[3] Martin J.C., Milliken D.L., Cobb J.E., McFadden K.L., Coggan A.R. (1998). Validation of a mathematical model for road cycling power. J. Appl. Biomech..

[4] Grappe F., Candau R., Barbier B., Hoffman M.D., Belli A., Rouillon J.D. (1999). Influence of tyre pressure and vertical load on coefficient of rolling resistance and simulated cycling performance. Ergonomics.

[5] Defraeye T., Blocken B., Koninckx E., Hespel P., Carmeliet J. (2010). Aerodynamic study of different cyclist positions: CFD analysis and full-scale wind-tunnel tests. J. Biomech..

[6] Blocken B., van Druenen T., Toparlar Y., Andrianne T. (2018). Aerodynamic analysis of different cyclist hill descent positions. J. Wind. Eng. Ind. Aerodyn..

[7] Grappe F., Candau R., Belli A., Rouillon J.D. (1997). Aerodynamic drag in field cycling with special reference to the Obree’s position. Ergonomics.

[8] Barelle C., Chabroux V., Favier D. (2010). Modelling of the time trial cyclist projected frontal area incorporating anthropometric, postural and helmet characteristics. Sports Eng..

[9] Beaumont F., Taiar R., Polidori G., Trenchard H., Grappe F. (2018). Aerodynamic study of time-trial helmets in cycling racing using CFD analysis. J. Biomech..

[10] Forte P., Barbosa T.M., Marinho D.A., Liu C. (2015). Technologic Appliance and Performance Concerns in Wheelchair Racing—Helping Paralympic Athletes to Excel. New Perspectives in Fluid Dynamics.

[11] Forte P., Marinho D.A., Barbosa T.M., Morais J.E. (2020). Analysis of a normal and aero helmet on an elite cyclist in the dropped position. AIMS Biophys..

[12] Heil D.P. (2001). Body mass scaling of projected frontal area in competitive cyclists. Eur. J. Appl. Physiol..

[13] Forte P., Marinho D.A., Morais J.E., Morouço P.G., Barbosa T.M. (2018). The variations on the aerodynamics of a world-ranked wheelchair sprinter in the key-moments of the stroke cycle: A numerical simulation analysis. PLoS ONE.

[14] Barbosa T.M., Ramos R., Silva A.J., Marinho D.A. (2018). Assessment of passive drag in swimming by numerical simulation and analytical procedure. J. Sports Sci..

[15] Barbosa T.M., Morais J.E., Forte P., Neiva H., Garrido N.D., Marinho D.A. (2017). Correction: A Comparison of Experimental and Analytical Procedures to Measure Passive Drag in Human Swimming. PLoS ONE.

[16] Blocken B., Defraeye T., Koninckx E., Carmeliet J., Hespel P. (2013). CFD simulations of the aerodynamic drag of two drafting cyclists. Comput. Fluids.

[17] Dorel S., Hautier C.A., Rambaud O., Rouffet D., Praagh E.V., Lacour J.-R., Bourdin M. (2005). Torque and Power-Velocity Relationships in Cycling: Relevance to Track Sprint Performance in World-Class Cyclists. Int. J. Sports Med..

[18] Aroussi A., Kucukgokoglan S., Pickering S.J., Menacer M. Evaluation of four turbulence models in the interaction of multi burners swirling flows. Proceedings of the 4th International Conference on Multiphase Flow.

[19] Faria E.W., Parker D.L., Faria I.E. (2005). The Science of Cycling: Factors Affecting Performance—Part 2. Sports Med..

[20] Heil D.P. (2005). Body size as a determinant of the 1-h cycling record at sea level and altitude. Eur. J. Appl. Physiol..

[21] Buchheit M. (2016). Chasing the 0.2. Int. J. Sports Physiol. Perform.

[22] Blocken B., van Druenen T., Toparlar Y., Malizia F., Mannion P., Andrianne T., Diepens J. (2018). Aerodynamic drag in cycling pelotons: New insights by CFD simulation and wind tunnel testing. J. Wind Eng. Ind. Aerodyn..

[23] Griffith M.D., Crouch T., Thompson M.C., Burton D., Sheridan J. Elite cycling aerodynamics: wind tunnel experiments and CFD. Proceedings of the 18th Australasian Fluid Mechanics Conference.

[24] Blocken B., Toparlar Y. (2015). A following car influences cyclist drag: CFD simulations and wind tunnel measurements. J. Wind Eng. Ind. Aerodyn..

[25] Bixler B., Pease D., Fairhurst F. (2007). The accuracy of computational fluid dynamics analysis of the passive drag of a male swimmer. Sports Biomech..

[26] Candau R.B., Grappe F., Menard M., Barbier B., Millet G.Y., Hoffman M.D., Rouillon J.D. (1999). Simplified deceleration method for assessment of resistive forces in cycling. Med. Sci. Sports Exerc..

